# “We no longer live in the old days”: a qualitative study on the role of masculinity and religion for men’s views on violence within marriage in rural Java, Indonesia

**DOI:** 10.1186/1472-6874-14-58

**Published:** 2014-04-16

**Authors:** Elli N Hayati, Maria Emmelin, Malin Eriksson

**Affiliations:** 1 Department of Public Health and Clinical Medicine, Epidemiology and Global Health, Umeå University, Umeå, Sweden; 2 Faculty of Psychology, Ahmad Dahlan University, Jl. Kapas 9, Semaki, Yogyakarta 55166, Indonesia; 3 Rifka Annisa Women’s Crisis Center, Jl. Jambon IV, Komplek Jatimulyo Indah, Yogyakarta 55241, Indonesia; 4 Department of Clinical Sciences, Social Medicine and Global Health, Lund University, Lund, Sweden

**Keywords:** Domestic violence, Masculinity, Positional map, Indonesia

## Abstract

**Background:**

Previous studies on domestic violence in Indonesia have focused primarily on women’s experiences and little research has been undertaken to understand men’s views on domestic violence or their involvement in the prevention of domestic violence. This study aimed to explore men’s views on masculinity and the use of violence within marriage, in order to gain knowledge on how to involve men in prevention of domestic violence in rural Indonesia.

**Methods:**

Focus group discussions with six groups of local male community leaders in Purworejo were conducted. The discussions were transcribed and coded for the construction of a positional map on different masculinities and their relation to the level of acceptance of domestic violence.

**Results:**

Social and cultural changes have played a crucial role in transforming the relationship between men and women in Indonesian society. Three different positions of masculinity with certain beliefs on the gender order and acceptance of violence within marriage were identified: the traditionalist, the pragmatist, and the egalitarian. The traditionalist had the highest acceptance of violence as a tool to uphold the superior position of men within marriage, while the pragmatist viewed violence as undesirable but sometimes needed in order to correct the wife’s behavior. The egalitarian did not see any reason for violence because they believed that men and women are equal and complementary to each other.

**Conclusions:**

Adaptation to social and cultural changes combined with lack of exposures to contextual and progressive religious teachings has led to the formation of three different positions of masculinity among the population in this study. Each position has certain beliefs regarding the gender order and the use of violence within marriage. Religion is an extremely important aspect that must be included in every type of intervention with this population.

## Background

The International Conference on Population and Development (ICPD) in Cairo in 1994 urged strongly that men become more involved in reproductive health and more engaged in efforts to eliminate violence against women. This international recognition was a result of an increased global awareness of the importance of sustainable development through education (especially for girls), gender equity, infant, child, and maternal mortality reduction, and the provision of universal access for reproductive health service including family planning and sexual health [1].

Most men are not violent, but when domestic violence occurs it is mostly men who are the perpetrators [2]. A study from Australia examined data from different agencies such as the police, courts, hospitals, and general medical practitioners that respond to victims of violence. The authors found that while there are certainly male victims of domestic violence the vast majority of victims are female [3]. Results from other studies suggest that constructions of masculinity play a crucial role in shaping men's attitudes toward violence against women. These constructs occur at the individual level, in families and relationships, in communities, and in societies as a whole [2]. Studies have found that men’s adherence to sexist, patriarchal, and/or sexually hostile attitudes is an important predictor of spousal abuse [4,5]. A literature review done by Moore and Stuart [6] on the relation between masculinity and violence found that men’s beliefs about appropriate male behavior, men’s appraisal of stress, challenges and threats toward masculine norms, and power imbalance in a relationship were predictive factors of domestic violence. Meanwhile, Heise [7] found that male domination in economic matters and in decision-making in the family is one of the strongest predictors of high levels of domestic violence against women.

Masculinity is a term that exists in a system of gender relations where men and women proceed a relationship in a gendered lives. According to Connell, masculinity is a relational concept that holds meaning in relation to femininity as a cultural and social demarcation [8]. Masculinity signifies a difference from femininity, and is derived from the concept of gender, which constitutes a set of characteristics and expectations on how men (and women) should behave within a given culture and time [9]. Because perpetrators of domestic violence are predominantly men, constructions of masculinity seem to play an important role in the occurrence of such violence [7]. Thus, it is vital to address men and masculinity in efforts to eliminate violence against women, including domestic violence. Despite international calls for action to involve men in ending violence against women in general and domestic violence in particular, most action and research have taken place in high-income countries such as the United States, the United Kingdom, Australia, and Canada [10]. Little is known about the link between masculinity and domestic violence in middle- and low- income countries with a predominantly Muslim population such as Indonesia.

Our previous population-based study among cohort of 765 reproductive age women in Purworejo District, Indonesia, revealed that the lifetime prevalence of physical violence was 11% and sexual violence was 22% [11]. The risk factors included husbands’ demographic characteristics such as lower education, younger age and wives’ characteristics such as economic independency and traditional attitudes toward gender relations [12]. A qualitative study among men in urban Yogyakarta and rural Purworejo found that there was a tendency toward ambiguity of self between being proud to be born to the superior sex that will become the leader in a household and an awareness of the burden he will bear due to that status [13].

In this study, we aimed to gain an understanding of men’s views on masculinity and violence within marriage in Javanese Indonesia.

## Methods

### Terminology

The term “violence against women” is based on the UN declaration that was launched in 1993 by the General Assembly entitled "The Declaration on the Elimination of Violence Against Women" (DVAW 2003). Since the launching of the declaration, violence against women perpetrated by an intimate partner within domestic life has become one of the most internationally highlighted topics within the field of violence against women. To date, diverse terms are used to name this type of violence such as wife assault/abuse, domestic assault/abuse/violence, women/wife battering, female partner abuse, and intimate partner abuse/violence depending on the type of intimate relationship such as current or former spouse, married or cohabitating couples, and heterosexual or homosexual couples. The term “domestic violence” is still used in many countries, including Indonesia, and in the UN bodies to refer to violence against women perpetrated by an intimate male spouse. Because “domestic violence” is the official term used in Indonesia, and due to the fact that this study focuses on violence within marriage life, we use the terms “domestic violence” and “wife abuse” interchangeably in this article.

### Study setting

Upon the ratification of the UN Convention on the Elimination of Discrimination Against Women (CEDAW), the government of Indonesia established a Ministry of Women’s Affairs (renamed in 2000 into the Ministry of Women Empowerment and Child Protection/MOWECP) to coordinate the implementation of equal opportunities for Indonesian men and women in the development of the country. This policy change has challenged cultural values for men and women and encouraged a shift in the nation from male dominated to a structure of greater gender equality. This has had significant implications for the sociopolitical and economic life of the country in terms of the relationship between men and women in society. However, the Marriage Law (Law number 1/1974) – which is still valid in 2014 – states that a husband is the head of the household and the wife is to manage the daily routines [14]. This law reflects gender labor division within marriage and is not aligned to the national policy on gender equality.

Under the new political reformation government in 2000, violence against women was officially declared to be a national problem and the Indonesian Domestic Violence Act (DV Act) was endorsed in 2004. Officially this law stated that domestic violence is *“any act toward somebody in the household, especially women, that results in any psychological, physical and/or sexual suffering, and/or abandonment, including threat, force, or deprivation of liberty as defined by law, that happened within the domestic sphere”* (Article 1). This statement confirmed that the Indonesian DV Act protects everybody living in the same household, including the wife, husband, children, relatives, and even domestic helpers who live in the household (Article 2) [15].

This study was conducted in Purworejo District, Central Java Province, which is located around 60 km west of Yogyakarta Province. According to the 2011 census, Purworejo District had a population of 696,141 people and a total area of 1,035 km^2^ including coastal, lowland, and hilly areas. Urban centers are found in the district, but 85% of the population lives in rural areas with farming as the major occupation [16].

In 2000, the district government formally appointed the Office of Social Affairs and People Empowerment to form a task force unit with the main function of providing a complaints desk for women survivors of domestic violence. In 2009, this office was renamed to the formal office for P2TP2A (*Pusat Pelayanan Terpadu Pemberdayaan Perempuan dan Anak*/Integrated Service Center for the Empowerment of Women and Children Survivors of Violence). One of the programs conducted by this office is to raise awareness of the problem of domestic violence and child abuse as well as of the legal aspects surrounding violence that occurs within the home.

### Study design

To answer our research question of how men’s views on masculinity affect violence within marriage, we chose to conduct Focus Group Discussions (FGDs) as the data collection method. FGDs allow researchers to utilize group interactions to explore people’s personal experiences and knowledge of a certain topic and are ideal for capturing experiences, opinions, and normative systems [17]. The advantage of using FGDs is their explicit utilization of group interactions to generate data and insights that would be less accessible without the interaction of people within a group [18].

### Sampling of informants

Six FGDs were conducted in six sub-districts in Purworejo. All of the male participants were local community leaders in their villages who had been selected by the village authorities to participate in the FGDs. The definition of a “community leader” was left to the village authorities to decide based on their knowledge of the local community. Two FGDs out of the six were attended by some college students who represented the “younger generation” and were considered by the people in those two sites to be “youth community leaders”. To understand more about the participants, we distributed attendance lists at the FGDs that requested the participants’ names, ages, educational levels, and occupations. The criteria for participation were: being an adult man and a local community leader. Since we wanted to get wide and rich information on the topic, we did not put any additional criteria for participation such as level of education, age, and occupation, etc.

### Data collection

To facilitate interactions and discussions, moderators of the FGDs were two male colleagues, one originating locally from Purworejo and one from Rifka Annisa, Yogyakarta (the Women’s Crisis Center based in Yogyakarta, around 50 km east of Purworejo). They were both trained in gender and gender-based violence issues so were knowledgeable in this field. Each FGD took around 1.5 to 2 hours, and all were recorded for subsequent transcription. Data collection took place from December 2007 - August 2009. The FGD guide included topics on the participants’ views on masculinity, men’s roles within marriage, and the husband’s use of violence within marriage.

### Analytical approach

**Figure 1:**
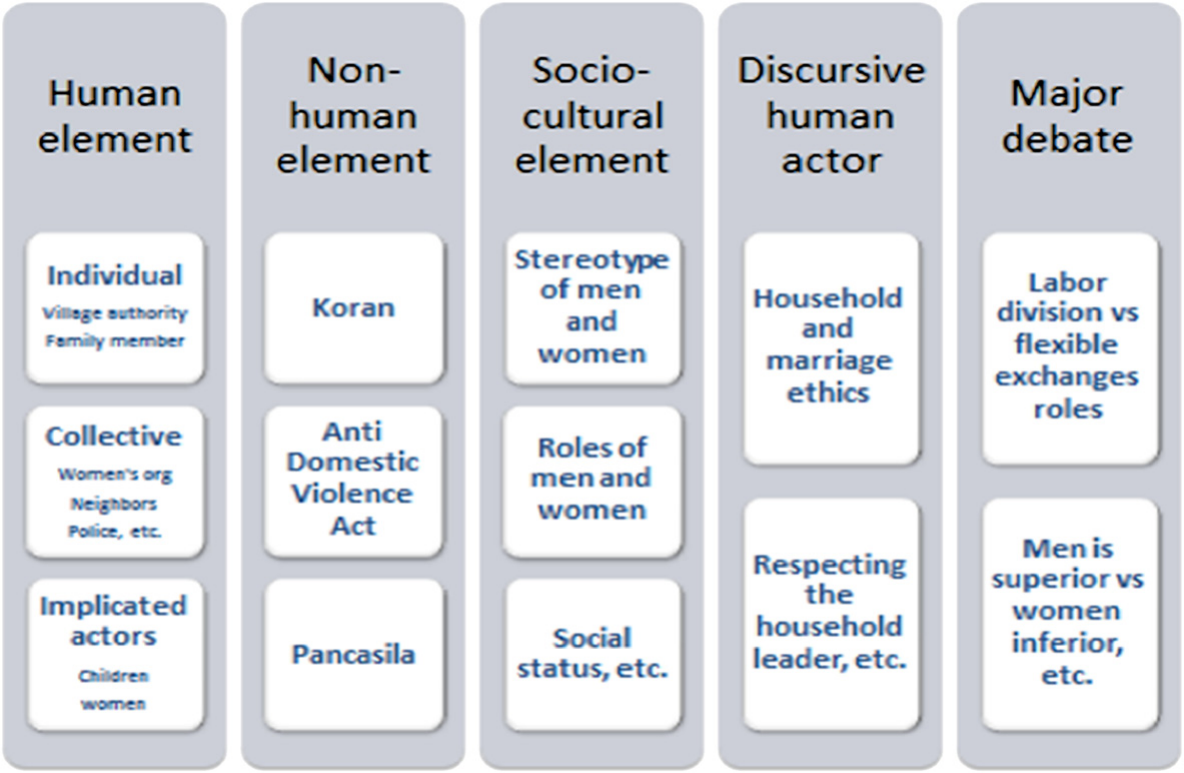
Part of the ordered situational map (OSM) identified in the initial analysis.

This study used Situational Analysis (SA) as proposed by Clarke [19]. SA is a further development of Grounded Theory, which was developed by Glaser and Strauss in 1967 [20]. The advantage of using SA is its focus of not only looking for a pure *"basic social process"* but also its ability to analyze power relations in a post-structuralist manner and to sufficiently reflect materiality*.* SA expands upon Grounded Theory by representing the field's messiness and complexity and by presenting more *reflexivity*, *uncertainty*, *modesty*, and *representation of contradictions*[19]. After critical readings of the transcripts, we manually performed a traditional Grounded Theory open coding and the open codes were put into a messy situational map (MSM). This MSM consist of codes (words or short sentences that reflect symbols or meanings of certain statement as written in the transcripts), of all the elements in the research situation, and was used as a basis for our further analysis. Through this first step, we started to identify the most relevant analytical elements related to men’s view on masculinity and violence within marriage. The identification of the most relevant elements resulted in the construction of an ordered situational map (OSM) that consisted of codes and categories of the major elements in the situation of inquiry, as suggested by Clarke [19]. The OSM contained 11 major elements related to men’s views on masculinity and violence, namely *Human elements* (*Individual, collective, and Implicated actors); Non-human elements/actants; Social cultural elements/symbos; Stereotype on man & woman elements; Economic elements; Political elements; Discursive human actors; Psychosocial elements; Preventive action elements; Spatial elements;* and *Major debate elements.* The two initial maps (MSM and OSM) helped to lay out the major human, nonhuman (e.g. things and particular knowledge/information influencing the situation being studied), discursive, and other elements in the research situation as a basis for further analyses. These maps are intended to capture and discuss the messy complexities of the situation in their dense relations and permutations [21]. Figure 1 illustrate the content of five identified major elements in the OSM that was developed from the raw messy situational map (MSM).

In addition, these initial situational maps helped us to further identify and analyze the major debate elements that were used as the basis for the axes of the final positional map. Positional maps lay out the different positions taken in the data regarding particular discourses of concern and the controversies and debates surrounding the subject being researched [19]. In this paper, we present the positional map to describe the final results of our analyses.

### Trustworthiness

To increase the trustworthiness of the study, the first author frequently visited the study site as part of a prolonged engagement with the local population. Regular peer debriefing sessions were also held within the research group to discuss the theoretical and conceptual basis used and to broaden the perspectives of the study. During data analysis, triangulation in terms of researchers was performed to discuss the interpretation of the analyzed data. The discussion rounds were stopped once the research team achieved agreement on the final interpretation of the data analysis.

### Ethical approval

Ethical approval was obtained from the ethical review board of the Faculty of Medicine, Gadjah Mada University, Yogyakarta and from the Purworejo District Government. Verbal and written consent was obtained from all participants prior to the FGDs. To maintain confidentiality, the moderators encouraged the participants to keep and protect each participant’s views, opinions, and expressions within the group and not to use these to judge or evaluate one another during or after the FGDs. Further, we also avoid writing the names of the villages where we conducted the FGDs and instead only indicate their locations geographically.

## Result

A total of 44 men that were identified as community leaders participated in six different FGDs each composed of 5 to 9 participants. Their ages ranged from 20 to 71 years, with a mean age of 39 years, and 88% were married. Other information on participant’s characteristic are listed in Table 1.

**Table 1:** Participants profile of the FGD

**Criteria**	**Category**	**Composition**	**Total**
**Education**	<9 years	17 (39%)	44
	>9 years	27 (61%)	
**Occupation**	Farmer	2 (4%)	44
	Civil servant	2 (4%)	
	Retirement	3 (7%)	
	Private employee	3 (7%)	
	Merchant	4 (9%)	
	Village authority	23 (52%)	
	Student	3 (7%)	
	Unspecified	4 (9%)	
**Village**	Lowland sub-district 1 (FGD 1)	7 men	44
	Coastal sub-district 2 (FGD 2)	8 men	
	Hilly sub-district (FGD 3)	7 men	
	Lowland sub-district (FGD 4)	8 men	
	Coastal sub-district (FGD 5)	5 men	
	Hilly sub-district (FGD 6)	9 men	

We present the results in two sections. The first results are related to the general pattern in the sociocultural environment of this study. This pattern reflects the perceptions of masculinity within marriage that are related to the changing sociopolitical environment in Indonesia. Secondly, we present a map of the positions taken by men that represent different views on masculinity and men’s role within marriage in relation to their potential acceptance of domestic violence.

### Masculinity in transition: the ideal husband vs. the real husband

From the elements that we identified in the OSM (partly shown in the paper, Figure 1), we found that religion is manifested in almost all elements (human elements, non-human elements, preventive action elements, psychosocial elements, spatial elements, and major debates). Thus it is obvious that religion is an extremely important aspect that must be included and embedded in every type of intervention to be developed in this site.

Next, from the OSM we found several codes describing strong sociocultural and economic elements related to religious- and cultural-based expectations of men and their roles in marriage. From the OSM, we also identified major debates related to masculinity in the marriage in terms of what constitutes the ideal man and the real man. All participants believed that the images of the ideal husband and ideal wife originated from the literal/textual interpretation of the Koran that is taught in their daily life. We found that these men were struggling to reorient their positions as men in relation to the current society governed by a transitional regime that had enacted rules on gender equity. This discrepancy between the ideal husband and the actual (real) husband was found to be the core theme in our data.

“The Koran is the basic rule on what the role of being a man is in the marriage. He is the leader and is responsible for fulfilling the family’s needs, the food, school fees, welfare…all of these are the man’s responsibility. This is given…it is natural…that man should be the breadwinner…the ideal man should be able to afford his household’s needs (FGD 1)”.

From the FGDs, it was clear that a man needs to have a stable income in order to manage his family well and to be able to avoid marital conflicts due to the economic hardship. We identified many codes describing economic elements related to men’s financial responsibility to uphold their position as a household leader, and these codes indicated the importance for men to have enough capital to reach social status as an honorable man. Education and job opportunities were seen as the main capital for men to achieve that status.

“Boys should be prioritized for school for the sake of their future roles as the responsible person in the family. It is a dignity to be born a man, and they will bear a big responsibility in life. That is why men get privileges as stated in the Koran, and why they get a greater share for their inheritance compared to women (FGD 3)”.

Even so, some talked about the different capacities among boys and girls in their academic achievements, attitudes, and daily manners in which girls are more successful in school than boys.

“I heard from the schoolmaster that girls perform consistently better than boys in terms of academic achievements…while boys…they cannot resist external temptations such as drinking or being preoccupied with PlayStations or games…that kind of thing. I noticed that the first rank students each year were always girls (FGD 1)”.

Furthermore, statements regarding women’s public achievement in comparison to men’s were also revealed. This was related to the current change in the state policy on the equal opportunities for women and men in participating in the country’s development.

“These days, more and more women go to universities and get well-paying jobs, sometimes their income is even higher than their male spouses’. Well…that is an unavoidable situation…the government has opened the opportunity for women to be employed as men, to be civil servants. We no longer live in the old days (FGD 5)”.

This shifting public recognition of men’s and women’s competency is unavoidable and has changed the job market dynamic in which men and women have become competitors in the public sphere. Some men are aware of their own shortcomings and flaws and admit women’s capability.

“I think men have more egotistical and fiery emotions, and if we look at those aspects then women are actually more capable of performing a job these days. In previous times, men were better but nowadays the abilities of men are not something to be proud of. If we look at education now, it is not only men who can become highly educated but women as well (FGD 2)”.

However, some participants opposed statements such as the one above, and referred to the Koran as the basic source of their views, i.e. that men were created to be superior to women.

“Even though women have shown their ability to get better jobs and positions, high salaries, and are able to fulfill the household’s needs on their own, still…they must admit that their nature is subordinate to men. Woman was created from man’s rib. Career is one thing, but nature is a necessity (FGD 3)”.

In general there was a gap between what these men believed to be the “ideal man” as taught by their religion, and the facts visible in society about what has been achieved by men (and women). These men were consistently exposed to religious teachings that led them to interpret the values of men and women textually, whereas at the same time they lived in a nation that endorsed gender equality. In our interpretation, this created a gap between an “ideal husband” as described in the Koran and a “real husband” living in a society with an overall gender equality policy. The sociopolitical and economic consequences of that gender equality policy have challenged men to reorient themselves as to what their positions are in relation to women in both marriage and in society.

### Three constructions of masculinity: *the traditionalist – the pragmatist – the egalitarian*

The above description describes the background of men’s ambiguity in how to position themselves, especially within private marriage life. This ambiguous situation has led to the construction of three masculine identities as determined from the FGD transcripts.

The first masculine identity was represented by codes talking about the importance of persisting in the beliefs that man is the superior sex and charged by God to be the leader and the decision maker (*the traditionalist*); the second was represented by codes indicating that man is the superior sex but that he has some flaws that can be covered by women (*the pragmatist*); and the third was represented by codes indicating that men and women are equal beings (*the egalitarian*). These “masculine identities” have somewhat different beliefs on how to set the relational system of man and woman within marriage, including the roles of husband and wives and views on wife abuse. At the end of this subsection we present a map of three different positions taken by men that represent different views on masculinity within marriage in relation to their potential acceptance of the occurrence of wife abuse.

### *The traditionalist* – “the more we empower the woman, the more she will disobey the husband”

This position represents a view that uses the text of Koran as the starting point in discussing man and his roles in marriage. According to this view, what the Koran says is completely valid and should be applied unquestionably to regulate all aspects of people’s lives. In terms of being a man, those who take the traditionalist position try to justify their superiority by presenting how the majority of key persons and public decision makers are men.

“All persons seated in this village structure are men. Recently some women have sat at the staff level as well, but just ordinary staff, you know, and only a few. In the national government, see…very few women are in ministerial positions. And what about the military platoons? All are men. All prophets are men, there are no female prophets…don’t you see that those all mean that men are better, that they have a higher value compared to women? (FGD 4)”.

Within this view, there is a belief that man’s supremacy is created and appointed by God, which implies that being a leader of women and being the head of the household is something irreplaceable.

“A man should have high self-esteem and struggle for his family life, and this is a way for a man to make it clear in the marriage that he is the leader, and that he will be respected by his wife. A good woman obeys her husband. If things were the other way around (the man was subject to the wife), that would mean that the man has no manhood (FGD 5)”.

Because the man in this view is the responsible person for earning a living, the woman is the one who must be led and can only be in charge as the responsible person for childcare and household maintenance. The woman should be able to manage her husband’s income to run the daily life of the family, and she is supposed to be grateful regardless of her husband’s ability in bearing the family life. In this sense, the division of roles is important in managing the family.

“While a man’s obligation is earning a living for his family, a woman’s obligation…reliable women are able to managing their husband’s income to run the daily life, caring for the kids, and serving their husbands…to greet him with a smile when the husband comes home…you know…and the husband does not need to be involved in household work…the women do (FGD 3)”.

The traditionalists are against the women empowerment programs that are encouraged by the government. They assume that women empowerment will only motivate women to oppose men and to search for public careers instead of caring for the family. They are also against the idea of women’s participation in the labor market because they feel it will only ruin the marriage rather than benefit it.

“A woman’s place is in the home, there is no need to work outside. Having a public career will only make it easier for women to have extra-marital affairs with other men…that is why there will be more women in hell and more men in heaven because they had been so succumbed (FGD 1)”.

According to this view, the trigger of marital conflict is an economic and financial issue. The husband’s failure in providing for the household needs might be criticized by the wife, and this can easily lead to fiery situation because the wife is pressuring the husband’s self-esteem.

“Most conflicts are due to economic hardship, when the husband fails to provide cash or food for the daily meal. The wife should be listening and showing her understanding and respect for the husband, but sometimes they pour cynical words onto him so that the quarrel starts and…bum bum…(the sound of slapping). This was unavoidable when his dignity was offended (FGD 6)”.

Violence is also seen as the husband’s way to correct the wife’s behavior when the wife is considered to be disobeying and not keeping with religious teachings regarding her duties within the marriage.

“Religious women know how to respect the husband, even if she has a higher degree of education or a higher job position. Violence occurs due to a woman’s disability in respecting and obeying her husband so that he needs to use violence to correct her (FGD 3)”.

In general, the traditionalist’s view on masculinity and its relation to marriage is one of subordination of the opposite sex and persistence to the belief in male supremacy. In this view, wife abuse is tolerable because a wife needs to be controlled and the husband has the responsibility and right to do it.

### *The pragmatist* – “we can listen to a woman’s opinion, but we do not have to obey her”

This position has a more “pragmatic” view on the relationship between men and women. This masculinity sees man as the superior, but his superiority is not a reason for him to be the dominant person in the family. This masculine view believes that the role of the wife and husband is to be complementary to each other in managing the family life with the man acting as the “mentor”.

“We believe in what God commands us through the Koran regarding marriage. Man is created to be the guardian of his family, to comfort and protect it from any shortages. But, in guiding the family the man should build a dialogue with the family members. He should be able to listen and should be patient enough to treat them so that things will be easier to manage (FGD 6)”.

This implies a more thoughtful use of the Koran that emphasizes man’s leadership with a spirit of patience and wisdom. Within this perspective, being a man requires having positive qualities to support his role as the family guardian.

“The man should be able to think systematically, to be charming and friendly with his kids, and to maintain his charisma so that he will be respected by the family members. To his wife, he should be able to talk…to negotiate things… (FGD 2)”.

Regarding the daily household management, the pragmatist masculinity believes that the division of roles is important, but that they are open to negotiation according to the situation.

“In most families, decision making is done by the man, but sometimes it needs to be done more equally…if there is a problem and the man needs to make a decision, he might need to discuss it first with his wife. We can listen to and respect a woman’s opinion, but this does not mean that we have to obey her…. We make the decisions because we are the responsible person in the family (FGD 2)”.

Economic hardship might also trigger a wife’s unfaithfulness by having an extramarital affair with another man who is more financially secure. Such a situation could also result in violent behavior if the man believes that he should behave firmly to maintain the stability of the marriage.

“Maybe the wife feels unsatisfied with the financial situation of the husband because she has her own job outside the home…this could complicate the relationship…she might become seen more as her husband’s male colleague with a more stable career, and the husband will burn with jealousy. In this case, a husband should be able to behave firmly in supervising his wife (FGD 3)”.

Overall, even though the pragmatist masculinity has more flexibility in viewing the relationship of men and women within marriage, the men who ascribe to this view still believe that a husband’s violence is acceptable as a means of correcting the wife’s behavior.

### *The egalitarian* – “we are not a better sex than women”

Just like the other two previous positions, the egalitarian masculinity also holds the Koran to be the basic reference on viewing the relationship between men and women within marriage. However, this position differs in that it admits men’s own flaws and inabilities in totally upholding their responsibilities within marriage. Those who hold this view on masculinity also admit that some women do perform better than men in public careers, and they are aware that the era of male supremacy has already passed.

“If we talk honestly, we are not a better sex than women. Usually, we are just pretending to be tough, manly…but that is just a trick to hide our weaknesses and imperfections. We tend to blame others, our wives…we were too cowardly to admit our weaknesses (FGD 5)”.

Regarding the division of household tasks, this masculine position believes that marriage is a joint effort between a man and a woman in achieving their family aspirations. Household responsibility, therefore, is shared and there is no strict or fixed division based on sex. They are aware of the changing times and admit that both men and women have equal opportunities for public participation. Women’s capability, therefore, should be utilized for the welfare of the family.

“We need to cooperate in managing the household…I usually do the laundry or wash the dishes. My wife has a job outside the home so I have to support her in managing the household tasks, which is also a man’s responsibility. Besides, these days we are no longer in a situation where our wives just stay home…times have changed and we should both cooperate to manage the household, our own family (FGD 3)”.

Regarding causes for conflicts and violence, the codes representing this masculine identity indicate that a man is able to play his role well in his relationship with his wife by being open hearted, cool headed, and able to manage his anger and not use his muscular strength to beat up his wife.

“We should avoid arrogant behavior and domination of our own families, our wives. If we believe that we are a leader, we should be aware that a good leader is willing to listen to his people. As a man, we have weaknesses just like our wives. We both have strengths and weaknesses, and we must, therefore, open our minds to their inputs or even their critiques. Anger will only damage our kids (FGD 2)”.

Overall, the emerging discourse in this study was one of different masculinities in the transitional sociopolitical situation in Indonesia. Each identity has different views on how masculinity should work within marriage in relation to the occurrence of domestic violence as part of the characteristic of masculine identity.

**Figure 2:**
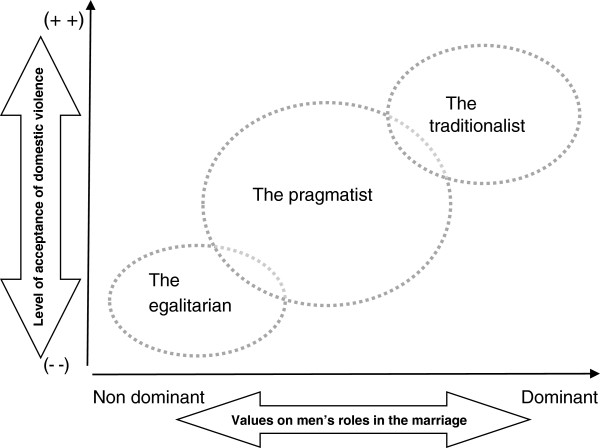
Positional map describing the values of men’s role in the marriage in relation to the level of acceptance of domestic violence.

Figure 2 illustrates how each masculine identity relates to the probability of wife abuse occurring within the marriage. *The traditionalist* beliefs that God assigns men as the superior ones, the leaders, while women are the subordinate ones, the led. The household role division is clearly set up under a single leadership: the husband. He is obliged to earn a living and fulfill the household needs, and the woman is obliged to manage the household routines, to bring up the children, and to serve and obey her husband. If she fails to meet that obligation, the husband may act harshly to uphold the rules. *The pragmatist* also believes that man is created by God as the superior one who is responsible for earning a living for the family and that women are obliged to manage the household and bring up the children. However, in this view men’s superiority is meant to be used wisely for the purpose of maintaining the family’s harmony. Therefore, the pragmatist supports flexible household rules between husband and wife and has an open mind for letting the wife participate in work outside the home and is willing to share the household roles. Even so, abusive behavior toward his wife is sometimes unavoidable as part of the husband’s leadership in supervising his wife. Thus, according to both the traditionalist and the pragmatist views, women are seen as responsible for violence when it occurs. The *egalitarian* also holds the Koran as their main reference. Despite this, they are open minded enough to admit that husband’s leadership is sometimes unbearable so that the husband needs to work hand in hand with the wife. In that sense, household division is not needed because the husband and wife are mutually sharing responsibility in the marriage. Wife abuse is avoidable because this masculine identity is not willing to dominate and is willing to have an open mind and admit each other’s strengths and weaknesses.

## Discussion

The FGDs revealed a general pattern in this study setting that implied a shifting of masculinity within marriage due to the changing sociopolitical conditions in Indonesian society. There were three different positions taken by men that represent different views on masculinity within marriage in relation to their potential acceptance of wife abuse. The different constructions of masculinity found in our FGDs were regardless of age of the male participants, since the unit of analysis refers to the group rather than individuals [17].

### The masculine incongruity

As a consequence of ratifying the UN CEDAW in the early 1984s [22] Indonesian society has undergone a very substantive transition from being a male-dominated society to becoming a gender-egalitarian society. However, the national Marriage Law still states that a husband is the head of the household and that the wife is responsible for maintaining the household. This guidance is deeply rooted in Islamic religious teachings on the roles of men and women in society and in the household. This contradiction between the socio-cultural and political background has become the source of ambiguity for the Indonesians, especially in applying the values of men and women in the daily practice of maintaining familial relationships.

As a consequence of the Indonesian ratification of the CEDAW, various specific new policies were implemented, one of which was the Ministry Of Women Empowerment and Child Protection that took the lead in coordinating the implementation of the CEDAW. This included policies targeted to improve women’s quality of life such as encouraging delaying the age at which one marries; reducing maternal mortality; increasing participation in education, the labor force, and economic and political spheres; limiting family size through independent family planning; and encouraging men to take a greater role in family life. The government’s efforts seem to be succeeding based on numbers in certain areas. According to United Nation for Children Fund (UNICEF), enrollment and attendance rates of boys and girls at primary school are the same, but in secondary school these rates are slightly higher for girls than for boys indicating no bias toward sons regarding access to education [23]. In addition, women’s participation in the job market has slightly increased from 51% of women participating in the labor force in 2005 to 55% in 2011. However, that number is far from men’s labor force rate of 85% in 2011 [24]. Even so, these numbers indicate that more women have entered the labor force and have become more independent economically. This also means that women’s participation within the household has diminished and that men are challenged to share their power as the main bread winner; to transition from being the only leader of the household into a mutual partnership; and to adjust from participating only in the productive public sphere to also participating in activities within the domestic sphere.

In relation to the national data, many statements by the men in this study, such as “we no longer live in the old days”, reflect an awareness of this transition and the challenges they face in re-positioning themselves within marriage and society. These men have been challenged to re-evaluate their positions within the context of marital relationships as a result of being exposed to two contradictory “doctrines” coming from different sources within the same nation. These different doctrines reflect the values of men and women according to religious teaching (man is the main bread winner, the leader, and the decision maker) and the values according to government policy (men and women are equal partners). The textual religious teachings on the values on men and women are relatively permanent in their interpretation (men are the leaders of women) because the Koran is considered as an eternal *syariah* (law) for humankind. Meanwhile, the government policy is constantly changing according to the nation’s development. Thus, the gender order is dynamic in society along with the technological and economic changes that demand a change in men’s role into a “modern” male role [25]. This situation has been causing ambiguity regarding the ideal self as a man according to the religious values and the real self as a man in the current sociopolitical climate.

Hacker [26] argues that the feeling of uncertainty and ambiguity regarding gender role expectations is reflective of an uncertain feeling toward the ability to fulfill or validate one’s manhood. Within the field of humanistic psychology, this situation is called as “incongruence”. This term was proposed by the prominent humanistic psychologist Carl R. Rogers who defined incongruence as a gap between the “ideal self” and the “real self” [27]. Rogers suggested that the incongruent individual tends to be defensive and cannot be open to different experiences and might even suffer from self-malfunctioning when facing difficult tasks and put under constant threat [27]. These individuals engage defense mechanisms such as distortion and denial, and this fact should be taken into account by policy makers who wish to find ways to narrow the gap between what it means to be an “ideal” man in Indonesian society and what it means to be a “real” man.

### Gendering violence

In relation to the adjustment to the change of external demands on gender requirements, our results suggest that men are “adjusting” themselves in three different ways. One way is to stick to the traditional view on men as superior, and thus the leader, and with a rigid gender role division within the household. According to Hacker [26], the massive social changes have not only affected the complementariness of the sexes, but also posed problems of personality fulfillment for men and women. Our results illustrate how the “traditionalist” position represents men who have lost the security of the old *paterfamilias* in which they were the autocrats of the breakfast table. Men taking this position experience difficulties in establishing new roles within their family lives [26]. The traditionalist position defends the use of violence toward the wife by referring to upholding God-given marriage rules. According to Haj-Yahia [28], the man who is the patriarch maintains power over women and children in the family and society. Therefore, dominance, power, and control largely determine the nature of interactions between men and women in general, and not just between husbands and wives. Anthropological studies in Indonesia have shown that attitudes and behaviors of Muslims toward gender and women’s issues are influenced by the combination of a patriarchal culture and a patriarchal interpretation of Islamic teachings that support a tolerance for domestic violence [29].

Persisting to traditional gender values is a serious problem for all men, not just battering men but ultimately for the entire male-dominated society [30]. Our results revealed that those who were able to adjust to the recent sociocultural challenges, and even become open minded enough to assume an egalitarian position with women, were less supportive of using violence in resolving marital conflicts. Traditional values that put man as the superior one in earning a living could lead men to shame and could threaten his masculine self-concept [31]. Our previous survey in this setting found that women with economic independence were almost two times more likely to be exposed to sexual violence from their husbands compared to women who were pure housewives [12]. Women’s economic independence is one form of the change in gender order because traditionally women have been economically dependent on their husbands. According to Anderson and Umberson [32], domestic violence perpetrators’ actions are shaped by structural changes in the gender order. These authors conclude that by gendering violence, the batterers not only reinforce their masculinity but also reproduce gender as dominance. The cultural depiction of the husband as the breadwinner has supported the greater rewards accorded to men in the workplace, legitimized male power within the family, and provided men with resources for demonstrating their masculinity [33].

With regard to the structure of power within marriage, the traditionalist maintains power at the husband’s hand while the egalitarian shares the power equally between husband and wife. From this perspective, power and control in a relationship has also been studied in relation to domestic violence. A study on married Korean couples in the US revealed that wife abuse was significantly greater in male-dominant compared to egalitarian couples [34]. Moore and Stuart conclude from their literature review on masculinity and domestic violence that power and control likely comprise one component of masculinity: men’s use of violence in a male dominant relationship serves to maintain power [6]. In patriarchal societies such as Indonesia— where the social structure of oppression exists [35], men are given the power and right to control women as they are the main breadwinner for the family. Kimmel [36] suggests that domestic violence might be a way for men to utilize power and control over women through “instrumental violence”, a violence that occur as a way of not to show how powerful a man is, but as a man’s frustration to his powerlessness [36]. Thus, men might use different tactics and behaviours to maintain their superior position, and as their expression of keeping power and controlling their partners. This finding is important when developing future recommendations for the government’s anti-domestic violence programs in the population in this study.

### Methodological considerations

The conceptual strategies in Situational Analysis allow us to capture the messiness in the field to gain an understanding of the complex problem of domestic violence. The dual use of the MSM and OSM during the early phase of data analysis has greatly helped in identifying the major debates within the situation of inquiry that could be used as the axes for the positional maps. A positional map is an appropriate way to visualizing different views on masculinities within marriage and their association with domestic violence.

The limited presence of the first author during the FGDs could be considered a limitation of this study. The author’s absence prevented her from observing the non-verbal expressions of the participants during the FGDs. The composition of FGDs was homogeneous in the sense that all men were considered as community leaders, but heterogeneous in terms of marital status and age. The presence of the unmarried men in some of the FGDs means that those men were expressing their purely normative views on marriage and not those based on their real experience of marriage. The age difference in the groups might possible have led the younger men to hold back their views in the presence of older men, thus limiting the views of younger men. However, the male moderators were skilled and experienced in facilitating the FGDs in this setting, and they ensured full participation of all FGD members by actively encouraging every participant to raise his opinion and by underlining how different experiences in life that leads to different equally important opinions. Further, our effort in maintaining the trustworthiness of the study through prolonged engagement, peer-debriefing, and triangulation during data analysis improved the trustworthiness of the study.

Since our aim was not to compare views on masculinities and violence between different age groups of men, we did not consider homogeneity in terms of age in the composition of the FGDs. Instead, we wanted to get a broad and comprehensive picture of how men in rural Indonesia – community leaders in various ages – view masculinity and domestic violence. Thus, we are unable to rule out whether there is an age pattern in the three different masculine identities found among men in this study. However, it is reasonable to believe that some masculine identity, such as the egalitarian, might be more prominent among young men. If so, this would indicate a transition of masculinities from one generation to another with a potential positive influence on the prevention of domestic violence. Further studies are needed in order to explore how different masculinities with various acceptance of violence are represented among various groups of men in Indonesia.

## Conclusions

The sociocultural changes with regard to gender equality policies, combined with a lack of exposure to contextual religious teachings, serve as the crucial but complex environment for the transition of the gender order in Indonesia. Adaptation to this external change has facilitated the formation of three different positions/constructions of masculinity referred to here as the traditionalist, the pragmatist, and the egalitarian. Each position has certain beliefs on the gender order and the use of violence within marriage. The traditionalist had the highest acceptance of the use of violence as a tool to uphold the superior position of men within marriage while the egalitarian did not accept any use of violence because they believed that men and women are equal and complementary to each other.

Based on the findings in this study, the local government of Purworejo could develop their future program for addressing domestic violence prevention by using these maps and focusing on the following:

It is obvious that religion is an extremely important aspect that must be included and embedded in every type of future intervention in this site, and the emergence of the egalitarian position among men in this site indicates that gender equality is possible to achieve in this population.

a. Conducting routine public lectures to increase community knowledge and awareness of domestic violence – especially to those in remote areas – using friendly local art as the media, pictorial banners or posters with popular and practical language. Public lectures, seminars and dialogues should include:

(i) Information on the Indonesian DV Act (Law number 23/2004), to familiarize the law for people in general, and to let them know that domestic violence is no longer a private issue, and that survivors are protected by the state. Those all need to be clearly communicated to people.

(i) Safety plan information for women and their children, in the form of flyers or brochures containing important numbers and addresses they could reach in case of emergency.

b. Encouraging the progressive religious male teachers to conduct public discussions on the contextual interpretation of Koran, rather than just a literal/textual interpretation, regarding egalitarian values of men and women.

c. The Government should provide pre-wed training for couples who are going to register their marriage. This training could contain the concept of a “healthy-egalitarian marital relationship”, as well as knowledge on the Domestic Violence Act.

## Abbreviations

ICPD: International conference on population and development; CEDAW: Convention on the elimination of discrimination against women; UNICEF: United nation children’s fund; UN: United nations; MOWECP: Ministry of women empowerment and child protection; DV Act: Domestic violence act; OSAPE: Office of social affairs and people empowerment; P2TP2A: Pusat Pelayanan Terpadu Pemberdayaan Perempuan dan Anak; FGD: Focus group discussion; SA: Situational analysis; MSM: Messy situational map; OSM: Ordered situational map.

## Competing interests

The authors declare that they have no competing interests.

## Authors’ contributions

ENH participated in the design of the study and the data collection, was responsible for the preliminary analysis, and drafted the manuscript. MEm contributed to the design of the study and the analysis and interpretation of the results and participated in the revision of the manuscript. MEr contributed to the analysis and interpretation of the results and participated in the revision of the manuscript. All authors have seen and approved the final version of the manuscript.

## Pre-publication history

The pre-publication history for this paper can be accessed here:

http://www.biomedcentral.com/1472-6874/14/58/prepub
